# Preliminary analysis of immune-related markers in thymic carcinoid

**DOI:** 10.1186/s12967-023-04751-x

**Published:** 2023-12-09

**Authors:** Jiaqi Zhang, Chao Guo, Lei Liu, Ke Zhao, Mengxin Zhou, Yeye Chen, Shanqing Li

**Affiliations:** Department of Thoracic Surgery, Peking Union Medical College Hospital, Chinese Academy of Medical Sciences and Peking Union Medical College, Beijing, 100730 China

**Keywords:** Thymic carcinoid, Immune-related microenvironment, PD-L1, VISTA

## Abstract

The immune-related microenvironment of thymic carcinoid has rarely been reported. We analyzed the expression of PD-L1 and VISTA, and the distribution of CD4^+^ T cells, CD8^+^ T cells and CD68^+^ macrophages in the thymic carcinoid by immunohistochemical staining, and showed the correlation between these markers and clinical survival, indicating the potential therapeutic prospects.

## Dear Editor,

Thymic carcinoid, especially atypical carcinoid, remains the most common of thymic neuroendocrine tumor, accounting for 2–5% of all thymic tumors [1]. The thymus itself plays an important role in the development of T lymphocytes. The co-stimulatory pathway of T cells, together with Programmed Death 1 (PD-1) and its ligand (PD-L), involves the formation of central and peripheral immune tolerance [2]. V-type immunoglobulin domain-containing suppressor of T-cell activation (VISTA), a negative checkpoint regulator, showed significant homology with PD-L1 and PD-L2. As an alternative treatment for most advanced malignant tumors, immunotherapy showed scarce application in thymic tumors, the immune microenvironment of thymic tumor ,especially carcinoid, remains unclear.

In this study, 20 samples of thymic carcinoid (including 1 typical carcinoid and 19 atypical carcinoids) surgically resected from the Department of Thoracic Surgery, Peking Union Medical College Hospital were obtained and used to explore the expression of PD-L1 and VISTA, and the distribution of CD4^+^ T cells, CD8^+^ T cells and CD68^+^ macrophages in the thymic carcinoid by immunohistochemical staining.

The median age at diagnosis of these patients was 41 (34–60) years old, and the median tumor diameter was 5.4 (2.2–9.7) cm. Five patients had ectopic adrenocorticotropic-hormone syndrome, and 3 patients were diagnosed as multiple endocrine neoplasia (type 1). Ki-67 index was reported in 19 patients, with a median value of 10% (3–15%). Nine cases exhibited the median nuclear divisions of 3/10 high power field (2–10/10 high power field). There were 7, 3, 7 and 3 cases in Masaoka-Koga stages I, II, III and IV, respectively. As of the follow-up date, 2 patients died and the rest survived.

**Fig. 1:**
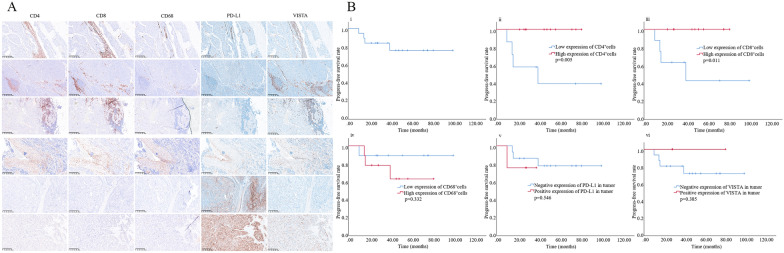
Immune microenvironment detection and prognosis analysis of thymic carcinoid. **A** Staining results of 6 samples with both PD-L1 and VISTA expression. **B** PFS analysis of patients with thymic carcinoid

The count of CD4^+^, CD8^+^ and CD68^+^ cells was 94 (21–340), 69 (23–475) and 75 (55–99) in each 20-fold field, respectively. Low- and high-expression groups were dichotomized by the median count of the immune cells. In terms of spatial distribution, CD4^+^ and CT8^+^ cells were mainly distributed in lymphocyte nests near tumor foci, while CD68^+^ macrophages were mainly distributed in the tumor stroma (Fig. 1A).

Positive PD-L1 expression (combined positive score ≥ 1 defined as positive expression) was presented in 8 cases (40%), of which 1 case was only expressed in tumor cells, 4 cases were only expressed in immune cells, and another 3 cases were expressed in both types. Positive VISTA expression (cell membrane or cytoplasmic staining of ≥ 1%) was presented in 11 cases (55%), of which 8 cases were only expressed in immune cells, and 3 cases were expressed in tumor cells and immune cells. Six cases (30%) expressed both PD-L1 and VISTA, as showed in Fig. 1A. Morphological observation showed that when CD4, CD8 and CD68 were highly expressed, the positive expression of PD-L1 and CD68 showed fusion regions, and the expression of VISTA and CD4 or CD8 positive cells showed similar fusion regions. The same pattern can also be seen in specimens expressing PD-L1 or VISTA alone.

Progression free survival (PFS) curves were drawn for 18 patients with thymic carcinoid, excluding 2 patients without definitive progression time (Fig. 1Bi). Univariate analysis showed that high-expression of CD4^+^ T cells (Fig. 1Bii, p = 0.005) or CD8^+^ T cells (Fig. 1Biii, p = 0.011) was significantly correlated with longer PFS. High-expression of CD68^+^ was seen to be prone to potentially shorter PFS (Fig. 1Biv, p = 0.332). Positive expression of PD-L1 (Fig. 1Bv, p = 0.546) or negative expression of VISTA (Fig. 1Bvi, p = 0.385) was correlated with shorter PFS. Although statistically differences have not yet been reached, it can be clearly seen from the survival curves between the two groups that the two curves have shown obvious separation, and the long-term survival status was stable. Perhaps due to the small sample size, the statistical difference still needs to be further explored. Liu et al. [3] showed that CD8 score may be negatively correlated with bone metastasis, and PD-L1 negative tumors were prone to lymph node metastasis.

In this study, morphological analysis of immunohistochemical results among different immune-related markers was conducted, and it was found that PD-L1 and CD68 showed a trend of co-expression. The PFS analysis results above showed that positive expression of PD-L1 in tumor and enrichment of CD68^+^ cells had a synergistic effect on patients’ PFS. The co-expression of PD-L1 with CD8 or CD68 has been thought to be associated with the efficacy of PD-1 inhibitors combined with chemotherapy in advanced non-small cell lung cancer [4].

The results similarly showed that VISTA had the same spatial distribution trend as CD4^+^ or CD8^+^ immune cells. Moreover, the results of PFS analysis showed that positive expression of VISTA in tumor and CD4^+^/CD8^+^ cells had the same influence on PFS. Previous studies [5] showed that VISTA was mainly co-expressed with CD68 in esophageal adenocarcinoma, while in the subgroup of tumor infiltrating lymphocytes, VISTA was mostly co-expressed with CD4 and was associated with long-term survival.

Above all, we investigated the expression and distribution of immune-related markers in the tumor microenvironment, suggesting the potential theoretical feasibility of immunotherapy in thymic carcinoid. By regulating immune checkpoints, the tumor-killing activity of immune cells could be mobilized, which may be a potential target of immunotherapy for thymic carcinoid in the future.

## Data Availability

The datasets used during the current study are available from the corresponding author on reasonable request.

## Abbreviations

PD-L1: Programmed death 1
VISTA: V-type immunoglobulin domain-containing suppressor of T-cell activation
PFS: Progression free survival
